# Prick tests and basophil activation tests in hypersensitivity to SARS-CoV-2 vaccines and components 

**DOI:** 10.5414/ALX02625E

**Published:** 2026-05-05

**Authors:** Anna-Emilia Aust, Heidi Reh, Bettina Wedi

**Affiliations:** Department of Dermatology and Allergy, Comprehensive Allergy Center, Hanover Medical School, Hannover, Germany

**Keywords:** allergy, anaphylaxis, basophil activation test, DSPC, vaccine, polyethylene glycol, polysorbate, prick test, SARS-CoV-2, trometamol

## Abstract

In collaboration with certified allergy centers (CACs) in Germany, a standardized procedure was developed for the allergologic evaluation of hypersensitivity reactions to SARS-CoV-2 vaccines. Our CAC prospectively evaluated 60 subjects with prior allergic reactions to SARS-CoV-2 vaccines or components. The investigations included a comprehensive medical history, in-vitro and skin tests. Prick tests were negative in all participants. Two positive basophil activation tests suggested a hypersensitivity to the component polyethylene glycol; however, no clinical relevance was found. Approximately 73% of the subjects reported symptoms during subsequent immunizations, which were milder than the reactions prior to the diagnostic procedures. Most index reactions manifested as non-specific symptoms. The results suggest that subsequent vaccinations following diagnostic procedures were well tolerated, underscoring the importance of careful allergological evaluation prior to vaccination.

## Introduction 

The first vaccine authorization in December 2020 marked a significant milestone in the effort to combat the SARS-CoV-2 pandemic [1, 2, 3, 4]. Initially, the mRNA-based vaccines Comirnaty (BioNTech, Mainz, Germany) and Spikevax (Moderna, Cambridge, MA, USA), as well as the vector-based vaccines Vaxzevria (AstraZeneca, Cambridge, England) and Jcovden (Johnson & Johnson, New Brunswick, NJ, USA) were available. The composition of the mRNA vaccines was based on a completely new method and contained additives that had not previously been used in vaccines or other injectable products. Initial clinical trials for the approval of mRNA-based vaccines demonstrated no cases of anaphylaxis. However, following the approval, cases of anaphylaxis have been reported, that have raised concerns not only among medical personnel but also among patients with a history of severe allergic reactions, atopic diseases, and other allergic manifestations [5, 6, 7, 8, 9, 10]. Initially, due to the paucity of data, neither the vaccine components responsible for the reported reactions, nor the underlying pathomechanism through which they were mediated could be identified [11]. Polyethylene glycols (PEG) were primarily suspected, given that immediate-type reactions, presumed to be IgE-mediated, had been reported previously [12]. Considering the medical necessity for repeated SARS-CoV-2 vaccinations, it has become increasingly clear that individualized recommendations for prospective administration of a specific vaccine are essential for individuals with existing or suspected hypersensitivity to vaccine additives. This statement also applies to people who have exhibited a hypersensitivity reaction to a specific SARS-CoV-2 vaccine in the past. 

Therefore, at the beginning of the vaccination campaign, several German allergy centers aimed to identify and phenotype hypersensitivity reactions to SARS-CoV-2 vaccine components using various allergological tests, including skin tests and, in selected cases, basophil activation tests (BAT). A total of 334 patients were included in these multicenter results, which have been published in an abbreviated form and only partially (e.g., only for certain vaccine components) [10, 13]. The results of this monocentric study, including the subsequent course following the allergological assessment, are presented in detail here. 

## Materials and methods 

### Patients 

From April 2021 to July 2022, a total of 60 study participants (aged 13 – 82) were prospectively recruited in the Department of Dermatology and Allergy at Hannover Medical School (ethics vote no. 9905_BO_K_2021). In the past, they had experienced an allergic reaction to medical products or vaccines containing ingredients such as polyethylene glycol (PEG, synonym macrogol), polysorbate (PS80, synonym Tween 80) or trometamol (synonym tromethamine, TRIS). The subjects were divided into two groups based on the index hypersensitivity reactions (Table 1). Group A (n = 44) exhibited an allergic reaction to a SARS-CoV-2 vaccine, while group B (n = 16) demonstrated a reaction to a component of the SARS-CoV-2 vaccines in another medical product. Supplemental Table S1 presents a comprehensive list of concomitant diseases and medications. 

**Figure 1:**
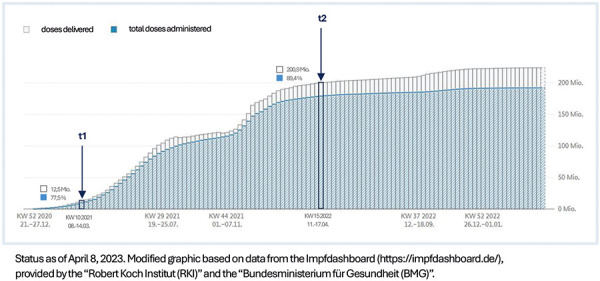
Availability of SARS-CoV-2 vaccines in Germany during the study period.

Figure 1 illustrates the temporal span of the study (t1: first patient for allergy diagnosis on March 12, 2021, t2: last patient on April 14, 2022) in relation to the availability of the various vaccines in Germany. 

### Medical history 

The medical history was collected using a comprehensive questionnaire developed by the Comprehensive Allergy Centers (CACs) of the lead CAC, Charité (Berlin, Germany). In addition to the general anamnestic information, participants were asked to provide details that would allow conclusions to be drawn about the type and time course of the symptoms of SARS-CoV-2 vaccine hypersensitivity. In order to ensure a consistent categorization of the reactions, they were divided into degrees of severity according to the classification by Ring & Messmer [14]. The time of onset of symptoms indicates an immediate or delayed type reaction [15]. All information was based on self-reporting by the participants and on medical reports from the attending physicians. 

### Skin testing 

The substances and concentrations used were carefully selected, considering the individual medical histories and the guidelines and recommendations that were valid at the time [11, 13, 16, 17]. Defined and titrated skin tests were performed in the form of a prick test (PT). At that time, higher molecular weight PEG (generally available with molecular weights from 200 to 35,000 kDa) and PS80 were the main focus of the suspected allergenic substances, which is why PEG2000, PEG6000, and PS80 were tested [18, 19, 20, 21]. These test solutions were freshly prepared as corresponding pharmaceutical-grade dispersion solutions in our in-house pharmacy, as there were no commercially available test materials. Allergic reactions to other SARS-CoV-2 vaccine components, such as 1,2-distearoyl-sn-glycero-3-phosphocholine (DSPC) and trometamol, were also recorded before the pandemic [22, 23], which is why these were also included in the study. The hospital pharmacy purchased the substances from Carl Roth, Karlsruhe, Germany (PEGs) and Merck, Darmstadt, Germany (PS80, trometamol, DSPC). However, only a limited number of allergological centers have performed skin testing with these additives [10]. The concentrations were chosen from stock solutions with 100 mg/mL as follows: PT at 1% and 10% [10]. The SARS-CoV-2 vaccines were tested directly pure and/or with a 1:10 dilution using sterile water as a dilutant depending on availability (brought in by participants or leftovers from staff vaccination campaigns at the clinic). A skin test was considered positive if an erythematous wheal ≥ 3 mm appeared after a reading time of 15 – 20 minutes [24]. Histamine solution was used as positive control and sodium chloride 0.9% as negative control. If the latter caused a wheal, the PT was considered non-assessable. 

### In-vitro diagnosis 

Total IgE, serum tryptase and specific IgE (sIgE) to latex were determined using ImmunoCAP (ThermoFisher Scientific, Waltham, MA, USA). In the BAT (FlowCAST, Bühlmann Laboratories AG, Schönenbuch, Switzerland) the stimulation was carried out with the available SARS-CoV-2 vaccine (1:10 and 1:100) and with PEG2000, PEG6000, PS80, DSPC, and trometamol (each 0.01%, 0.1%, 1%, and 10%) as well as additionally with commercially available DMG-PEG2000 from the company Bühlmann Laboratories AG (each 4 mg/mL: 1:2500, 1:500, 1:100 and 1:10), PEG2000 and PEG4000 (each 4 mg/mL: 1:100 and 1:10). Direct testing of all SARS-CoV-2 vaccines, or in some cases only the suspects, was not always feasible due to the acute vaccine shortage at the time of testing. An antibody against the high-affinity IgE receptor, FcεRIα, was used as a specific positive control and formylmethionylleucophenylalanine (fMLP) was used as a non-specific positive control. BAT was considered non-assessable if there were less than 15% activated basophils (CD63+/CCR3+ cells) with the positive control or more than 5% activated basophils with the negative control. 

### Recommendations and follow-up questionnaire 

Following the allergological workup, a personalized recommendation for the prospective vaccination protocol was issued. If there was evidence of sensitization to SARS-CoV-2 vaccine components, it was considered whether the benefits of vaccination outweighed the risk of an allergic reaction and whether a specific vaccine should be preferred in the selection process. Furthermore, preventive intake of antihistamines was recommended in some cases to prevent or minimize possible symptoms. If the patient agreed to be contacted again with the written consent for the study, details of initial or follow-up vaccinations were collected in writing or by telephone using a questionnaire designed specifically for this purpose. Reported symptoms were categorized into symptoms of an allergic reaction and atypical symptoms to distinguish hypersensitivity reactions from regular side effects. 

## Results 

### Initial allergic reactions prior to study inclusion 

In group A (n = 44) (Supplemental Figure S1), 40 participants reacted to an mRNA-based SARS-CoV-2 vaccine (n = 36 Comirnaty, n = 4 Spikevax). In contrast, only 4 participants reacted to a vector-based SARS-CoV-2 vaccine (AstraZeneca). Symptoms occurred predominantly within the first hour post-injection: within 0 – 10 minutes (n = 22), 11 – 30 minutes (n = 14), and 31 – 59 minutes (n = 4) (Supplemental Table S2). Few participants (n = 5) reported a delayed onset of symptoms (2 – 6 hours post-vaccination). Treatment mostly involved antihistamines, corticosteroids, volume administration, and intramuscular adrenaline. Some participants (n = 9) did not require any medication. 17 individuals were hospitalized due to the hypersensitivity reaction, with 4 participants receiving intensive medical care. Reactions primarily occurred in severity grades I to II according to Ring & Messmer. Severity grade III was registered in only 4 cases. 

Details of reactions to non-SARS-CoV-2-preparations in group B (n = 16) are presented in Table 2. 

### Diagnostic results of allergological workup 

**Figure 2:**
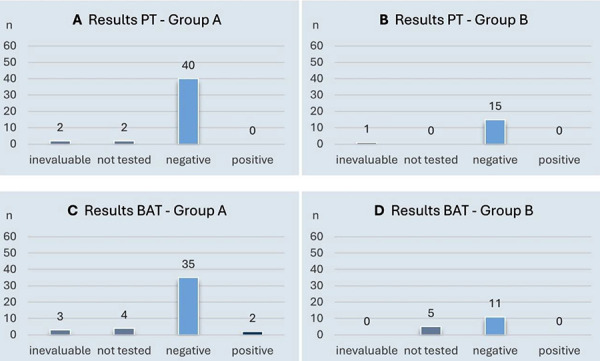
Results of allergological diagnostics using prick test and basophil activation test.

The titrated PTs with the vaccines as such or their components were negative in all participants of both groups (Figure 2A, B). However, 2 participants in group A showed a suspected hypersensitivity reaction to SARS-CoV-2 vaccine components by a positive BAT to DMG-PEG2000 or PEG2000 (Figure 2C, D) (Supplemental Table S3). 

In person 1 the index reaction was triggered by the Moderna vaccine, which also contains DMG-PEG2000. The index reaction of person 2 was triggered by a vaccine that contained neither DMG-PEG nor PEG2000, but PS80, suggesting a false positive BAT. Reported reactions to multiple PEG2000-free drugs further argue against PEG2000 hypersensitivity. Still, BAT results were positive for the PEG2000-containing Cominarty vaccine, while the PT was unremarkable. Consequently, avoidance of PEG- or macrogol-containing products, including mRNA-based vaccines, was recommended as a precautionary measure. As an alternative, immunization with a vector-based vaccine (Vaxzevria or Jcovden) was advised. The subsequent survey revealed a re-exposure in both cases due to the administration of the previously recommended vaccines Vaxzevria (n = 1) and Jcovden (n = 1). The latter caused a delayed symptom onset (6 – 24 hours post-vaccination), limited to usual side effects of vaccination. 

Total IgE and tryptase showed comparable results in both groups (Supplemental Table S4). 15.9% of the participants in group A and 18.8% in group B had an elevated total IgE value (> 100 kU/L). Elevated serum tryptase values (> 11.4 µg/L) were only found in 3 participants in group A with respective values of 15.5 µg/L, 15.6 /µg/L and 18.8 µg/L. 

### Re-evaluation of follow-up vaccinations 

41 out of 44 participants in group A (91.7%) took part in the questionnaire feedback. Of these, 34 people (83%) received at least 1 further SARS-CoV-2 vaccination after diagnosis. In group B, 14 out of 16 participants responded, with 79% of participants receiving at least 1 further vaccination. Details of SARS-CoV-2 vaccination doses administered after the allergological diagnostic workup are described in Supplemental Figures S1 and S2. 

Around three-quarters of respondents in both groups (group A: 73.5%, group B: 72.7%) reported symptoms during the follow-up vaccinations (Supplemental Table S5). Information on possible reinforcing factors such as physical or psychological stress, sleep disorders, infections, menstruation, or alcohol was only rarely provided, meaning that this data was not included in the results. While 47.1% of respondents in group A took pre-medication (antihistamines) before the vaccination, only 1 person in group B did so. In both groups, the predominant symptoms reported were skin and mucosa symptoms, in addition to other symptoms. The latter mainly included non-specific symptoms of common vaccination side effects such as headache, muscle pain and weakness, as well as fever. Furthermore, a large number of respiratory symptoms were recorded in group A. Compared to the reactions prior to the allergological diagnostics, milder degrees of severity were largely determined for the follow-up vaccinations based on the information provided by the study participants. The evaluation showed that medical consultation to treat the symptoms was only rarely required. Of all participants, only 4 people (group A) were hospitalized, 2 of whom planned their hospital stay in advance as a preventive measure to receive the vaccination under increased emergency preparedness. 

## Discussion 

### Female predominance in allergic reactions to SARS-CoV-2 vaccines 

Our data shows a pronounced predominance of female participants (90%) experiencing suspected allergic reactions to a SARS-CoV-2 vaccine or its components, compared to male participants (10%). This disparity was most evident in group A (95% females) compared to group B (75% females). These findings suggest gender-specific differences that may reflect underlying immunological mechanisms or differences in symptom perception and reporting, rather than group-specific characteristics alone. This observation is consistent with previous data reported by Lee et al. [25], who described a higher frequency of reported drug allergies in females in general, rather than specifically to vaccines. A study from Trieste found that 79.6% out of 269 individuals with suspected vaccine-related allergic reactions were female [26]. Further, data from the Paul-Ehrlich-Institute showed that women reported anaphylactic reactions more frequently than men after the first dose of mRNA vaccines (Comirnaty and Spikevax) and more frequently than women who had already received a second or third dose. Reported rates of anaphylaxis in women who had received their first dose of Comirnaty (BioNTech/Pfizer) or Spikevax (Moderna) were 0.97 and 1.12 reports per 100,000 vaccinations, respectively [27]. Taken together, our data reinforces existing evidence of gender-specific differences in reactions to SARS-CoV-2-vaccinations. This consistent observation across different study cohorts highlights the need for further research on this topic. 

### Challenges in interpreting self-reported symptoms following SARS-CoV-2 vaccination 

Interpretation of symptoms following SARS-CoV-2 vaccination is challenging, as data were primarily obtained through self-administered questionnaires and symptoms could not always be clinically verified by healthcare professionals. While objective symptoms can be confirmed by clinical examination or diagnostic tests, symptom perception varies individually. Of 117 symptoms reported, 53.85% were considered objective and 46.15% subjective. However, even objective symptoms are not necessarily indicative of allergic reactions and require careful individual evaluation. Symptom perception may therefore be influenced by individual circumstances, psychological comorbidities, and other variable factors. Several symptoms observed in our study may be stress-induced rather than caused by an actual allergic reaction to vaccine components. Desai et al. [28] demonstrated an association between psychological comorbidities and SARS-CoV-2 vaccine hesitancy, highlighting the role of psychological factors in the perception and reporting of vaccine-related reactions [28]. Common post-vaccination complaints such as nausea, headache, injection-site pain, cardiovascular symptoms, numbness, tingling, and restlessness are considered non-specific and not exclusive to allergic reactions [29]. According to the World Health Organization (WHO), many of these symptoms are among the most common stress-induced symptoms [30]. Without comprehensive clinical and allergologic evaluation, causal attribution to identify whether the symptoms are allergic or non-specific in origin remains uncertain. These aspects should also be considered when communicating about vaccination to mitigate hesitancy and enhance confidence in vaccination programs. 

### Reported symptoms following SARS-CoV-2 vaccination compared with previous studies 

This study demonstrates a clear difference in symptom distribution between groups A and B following SARS-CoV-2 vaccination after allergological assessment (Supplemental Table S5). In group A, respiratory symptoms predominated (97.2%), followed by skin/mucosal symptoms (53.2%) and cardiovascular symptoms (8%), while other non-specific symptoms were reported in 98.1%. Group B mainly reported non-specific symptoms (92.3%) and fewer skin/mucosal symptoms (44.4%) with no respiratory or cardiovascular involvement. This pattern suggests that group A is likely to reflect clinically relevant allergic reactions, whereas symptoms in group B are more likely non-specific or stress related. Thus, the considerably smaller number of participants in group B limits the reliability of direct comparisons. 

Comparison with the literature reveals both similarities and differences. In the multicenter study conducted by Worm et al. [10], where our study results have been incorporated, albeit in abbreviated form, the most frequently reported symptoms are as follows (reports of 219 participants): skin/mucosal symptoms (52.2%), cardiovascular symptoms (32%), respiratory symptoms (24.2%), and gastrointestinal symptoms (7.8%). Similarly, data from the Paul-Ehrlich-Institute [27] reported predominantly skin/mucosal reactions (45.9%), with respiratory symptoms (27.7%), cardiovascular reactions (14.1%), and gastrointestinal complaints (12.3%) occurring less often in 321 cases with confirmed allergic reactions. Pignatti et al. [31] likewise observed mainly skin/mucosal reactions (80%), while respiratory symptoms (24%), cardiovascular reactions (5%), and neurological or gastrointestinal complaints (5 – 7%) were comparatively rare in a total of 89 participants. 

Overall, our findings correspond with published data regarding symptom patterns and rapid onset after SARS-CoV-2 vaccination. While our own study shows a higher proportion of respiratory symptoms, skin/mucosal symptoms have been documented more frequently in other studies. This discrepancy may reflect cohort-specific characteristics or comorbidities and warrants further investigation. 

### Methodological differences in skin prick test and basophil activation test 

Despite early concerns that even allergologic testing might provoke severe reactions [13, 32], positive BAT results were observed in only 2 individuals and BAT results were not congruent with PT. These findings indicate that objectively detectable sensitization to vaccine components is rare and that BAT positivity is exceptional in this clinical setting. 

This study provides the first systematic data on trometamol and DSPC in allergologic diagnostics at that time. At the applied concentrations, neither substance induced irritative or false positive reactions in PT or BAT. These results were not included in the multicenter analysis by Worm et al. [10], as these additives were tested only in a few centers, including ours. At the time of our evaluation, there was only 1 case report of anaphylaxis to a gadolinium-containing contrast agent with trometamol [23]. There was no data on DSPC, which had hardly been used before. 

Consistent with our findings, previous studies [26, 27, 33] reported predominantly negative PT results when testing SARS-CoV-2 vaccines or their components. More than 75% of PTs were negative, with positive reactions occurring in fewer than 25% of cases. In these studies, diluted solutions of PEG (e.g., PEG3350 at 1 : 100, 1 : 10, 1 : 1) were used [26, 27, 33]. However, due to limited availability, additives actually contained in the vaccines were often not tested. Instead, PEGs of different molecular weights or unrelated PEG-containing drugs were tested. This heterogeneity limits direct comparability across studies. 

Data on BAT use in this context remain limited. While standardized allergens from Bühlmann Laboratories AG (PEG2000, PEG4000, DMG-PEG2000) have been used, many studies preferentially relied on PEG derivatives such as PEG3350 for in-vitro testing [31, 34, 35]. BAT using whole vaccines, trometamol or DSPC has been performed only rarely. Differences in allergen source, concentration and test target (pure additives vs vaccine formulation) must therefore be considered when interpreting diverging BAT positivity rates. 

In contrast to our low BAT positivity, Pignatti et al. [31] reported positive BAT results in 37% of patients with a prior reaction to a SARS-Cov-2 vaccine, predominantly directed against DMG-PEG2000 (Avanti, Alabaster, AL, USA, at 5 µg/mL and 0.5 µg/mL). Yet, re-exposure was well tolerated with over 93 – 100% in both the test and control group, including individuals with positive BAT results to DMG-PEG2000 [31]. Methodological differences, particularly the use of specific PEG-lipids and different test concentrations, may partly explain the higher BAT reactivity reported. 

Similarly, Jover Cerdá et al. [34] observed 100% negative PT results but frequent positive intradermal tests with a SARS-CoV-2 vaccine (1 : 1 and 1 : 10), while most patients subsequently tolerated revaccination, with or without premedication [34]. These findings further question the predictive value of positive allergologic test results. 

### Clinical implications 

Across studies, including our own, tolerance to revaccination despite positive PT or BAT results has been reported frequently [31, 34]. Accordingly, the diagnostic reliability of the BAT, particularly when positive, remains uncertain. Labella et al. [36] suggested BAT with PEG2000 as potentially useful for confirming PEG hypersensitivity but emphasized that BAT using a pure vaccine is unlikely to detect true hypersensitivity reactions and may instead reflect prior SARS-CoV-2 infection [36]. 

Due to the paucity of data regarding the tolerability of subsequent vaccinations in the event of positive test results, cautious recommendations for re-exposure were initially made at the outset of our study, in conjunction with avoidance. Based on current findings, re-exposure is currently advised in most cases under special precautions (emergency preparedness measures) obviating the necessity for prior allergological evaluation. Given current knowledge, reliance on positive BAT results alone to guide vaccine selection is not justified and may inadvertently expose patients to alternative vaccines with comparable or more severe adverse side effect profiles. 

In our cohorts, symptoms after re-exposure were predominantly subjective, non-specific and indistinguishable from a stress-induced reaction. Together with data from other centers, our results support the conclusion that true allergic hypersensitivity to SARS-CoV-2 vaccines is exceedingly rare and that the diagnostic value of positive BAT results is limited. The initial hesitancy related to SARS-CoV-2 vaccination could not be medically confirmed and has now lost its intensity. 

Notably, most patients proceeded with at least 1 additional SARS-CoV-2 vaccination, underscoring the significant benefit of allergological evaluation in reducing fear of allergic reactions. Further, experience during the pandemic demonstrated that PEG2000-containing SARS-Cov-2 vaccines are generally tolerated even in individuals with known hypersensitivity to PEGylated drugs [37]. In our group B, no positive test result or hypersensitivity reactions were observed after subsequent vaccination. 

### Advantages and limitations of the work 

This prospective study used standardized PT and BAT protocols. This method allowed a systematical analysis of all potential additives, including not only PEG2000, but also PEG6000, DSPC, and trometamol. In addition, several concentrations of these additives were tested within the PT and BAT, complemented by commercially available PEG allergens such as PEG4000 and DMG-PEG2000. The study was based on a monocentric collective with a high number of participants. Another positive aspect is the high response rate of the participants: 91.7% of the participants could be followed up. It is particularly noteworthy that 82% of these people tolerated another SARS-CoV-2 vaccination without any problems. 

Limitations include occasional unavailability of the suspected vaccine for testing. Moreover, the 2 BAT-positive patients did not undergo a provocation test due to their assessment occurring at the study’s inception, when a cautious approach was adopted. 

## Conclusion 

From an allergological perspective, SARS-CoV-2 components are not considered to pose a significant problem. The majority of the index reactions observed in this study are likely to have been non-specific symptoms such as palpitations, dizziness, or nausea. A detailed patient history of past illnesses and concomitant factors (e.g., physical or psychological strain, sleep disturbances, stress, infection, menstruation, alcohol) on the day of a vaccination is recommended for identifying and reassuring patients at risk in advance. Anaphylactic reactions in general, and in particular those following the administration of SARS-CoV-2 vaccines, are still considered to be rare [27]. The results showed that subsequent vaccinations following a diagnosis were, in general, well tolerated. These findings underline the importance of a careful allergological evaluation prior to vaccination, thereby contributing to the enhancement of safety and acceptance of SARS-CoV-2 vaccinations. In view of the ongoing vaccination campaigns, the obtained results are of particular significance. The additives under investigation – including PEG2000, PS80, DSPC, and trometamol – play a role both in the SARS-CoV-2 vaccines currently in use (Supplemental Tables S6 and S7) and in a variety of new therapeutic approaches, such as mRNA-based anti-tumor therapies and the use of lipid nanoparticles [38, 39]. 

## Acknowledgement 

We would like to thank the team at the in-house Hannover Medical School pharmacy, as well as the entire allergology team and the laboratory at our clinic. Their support and expertise made it possible to carry out this extensive allergological diagnosis. 

The English translation of this manuscript was generated with DeepL Pro and subsequently checked and edited for accuracy. 

## Authors’ contributions 

Conception and design of the study: Who was responsible for the idea and design of the study? AEA, BW. 

Data collection: Who collected the data or conducted the experiments? AEA, HR. 

Data analysis and interpretation: Who analyzed the data and interpreted the results? AEA, BW. 

Manuscript drafting: Who wrote the manuscript and who reviewed it? AEA, BW, in part HR. 

## Funding 

Performance-based allocation of funds by Hannover Medical School. 

## Conflict of interest 

AEA: no conflicts of interest. HR: no conflicts of interest. In the past 3 years, BW has received honoraria for lectures and advisory board meetings and/or funding for travel and attendance at congresses from the following companies: ALK-Abelló, Bencard, Biocryst, CSL Behring, HAL Allergy, Kalvista, Novartis, Takeda and ThermoFisher Scientific. 

**Table 1.** Comparison of the characteristics of the two study groups.

**Demographic characteristics**	Group A*	Group B**
Number of persons	44	16
Age range (in years)	16 – 82	13 – 69
Average age (in years)	45	55
Gender	95% female 5% male	75% female 25% male
Profession in the healthcare sector	25%	25%

*Allergic reaction to SARS-CoV2 vaccine; **allergic reaction to SARS-CoV-2 vaccine component / other medical product.

**Table 2.** Group B (allergic reaction to SARS-CoV-2 vaccine component / other medical product).

	**Administered substance**	**Additive (suspected allergen)**
Vaccine	Pandemrix	Polysorbate 80
	Encepur	Trometamol
	Influsplit Tetra	Polysorbate 80
	Unknown vaccine	
Chemotherapeutic agent	Paclitaxel	Macrogol glycerol ricinoleate-35
	Pegylated asparaginase	Monomethoxy polyethylene glycol
Laxative	Klean-Prep	Polyethylene glycol 3350
	Unknown laxative	
Other medication	Sympal	Polyethylene glycol 6000
	Ustekinumab	Polysorbate 80
	Unknown drug	
Contrast agent	Unknown contrast agent	

## Supplemental material

Supplemental material Supplemental Tables and Supplemental Figures.
