# Supplementary material for: Prick tests and basophil activation tests in hypersensitivity to SARS-CoV-2 vaccines and components
Source: Allergol Select. 2026 May 5;10:63–72. doi: 10.5414/ALX02625E (PMC13151903; doi:10.5414/ALX02625E)

Supplementary Tables

Suppl. Table S1 Concomitant diseases and concomitant medication of the two study groups

Concomitant diseases	Group A*		Group B**	
	Number (n)	Percent (%)	Number (n)	Percent (%)
Allergic rhinitis	12	27	4	25
Asthma	8	18	2	13
Atopic dermatitis	2	5	0	0
Cardiovascular disease	9	21	0	0
Chronic infection	1	2	0	0
Chronic obstructive pulmonary disease	1	2	0	0
Malignant disease	5	11	0	0
Mastocytosis	1	2	0	0
Nasal polyps	3	7	1	6
Thyroid disease	8	18	2	13
Chronic urticaria	2	5	0	0
Other illness	10	23	6	38
**Concomitant medication**	**Group A***		**Group B****	
	Number (n)	Percent (%)	Number (n)	Percent (%)
Angiotensin-converting enzyme inhibitor	6	14	0	0
Beta-Adrenoceptor Antagonist	5	11	1	6
Non-Steroidal Anti-Inflammatory Drug	8	18	1	6

* allergic reaction to SARS-CoV2 vaccine
** allergic reaction to SARS-CoV-2 vaccine component / other medical product

**Suppl. Table S2** Symptoms after injection - before allergological diagnostics		Group A*	
		Number (n)	Percent (%)
Airway	Hoarseness	2	3,5
	Tightness in the throat	17	29,8
	Persistent dry cough	2	3,5
	Swelling of the upper airways	17	29,8
	Stridor (inspiratory)	1	1,8
	None of the above	18	31,6
Breathing	Breathing difficulties without rales / stridor	12	26,7
	Shortness of breath / breathing difficulties	2	4,4
	None of the above	31	68,9
Shortness of breath Details	Tachypnoea	1	2,2
	Increased use of the auxiliary muscles	1	2,2
	None of the above	44	95
Cardiovascular system	Documented hypotension	4	8,5
	Shock	5	10,6
	None of the above	38	80,9
Shock Details	Tachycardia	3	6,5
	Clouding of consciousness	3	6,5
	No shock	40	87,0
Skin / mucous membrane	Urticaria limited to injection site	1	1,7
	Generalized tingling sensation	11	18,3
	Generalized itching without rash	5	8,3
	Generalized angioedema	1	1,7
	Localized angioedema	4	6,7
	Generalized erythema	14	23,3
	Generalized urticaria	8	13,3
	None of the above	16	26,7
Other symptoms	Headache	3	6,3
	Muscle pain and weakness	4	8,3
	Nausea	4	8,3
	Abdominal cramps	1	2,1
	Diarrhea	2	4,2
	None of the above	34	70,8

* allergic reaction to SARS-CoV2 vaccine

Suppl. Table S3 Overview - Participants with a positive result in allergological diagnostics (BAT)

Medical history	Person 1	Person 2
Gender	female	female
Age	42 (as of 01 October 2022)	31 (as of 01 October 2022)
Intolerances	tomatoes, apples, tree nuts/peanuts, soya; tingling sensation, oral swelling and diarrhea known when consumed	Penicillin, Paracetamol
Concomitant diseases	Allergic rhinitis	Allergic rhinitis<br>Bronchial asthma<br>Atopic dermatitis<br>Lupus erythematosus<br>Thyroid disease<br>Atransferrinemia
Medication	no medication	Pantoprazole<br>Morphine<br>Naproxen<br>Novamine sulfone<br>Pregabalin<br>Foster spray<br>Cerazette
Vaccine with reaction		
Vaccine manufacturer	Moderna	AstraZeneca
Date	20.05.2021	20.02.2021
Location	vaccination center	vaccination center
Type of vaccination	first vaccination	first vaccination
Known reaction		
Time until appearance	11-30 minutes after injection	0-10 minutes after injection
Symptoms	redness, itching	itching, urticaria, tightness in the throat, shortness of breath, unconsciousness
Severity (Ring-Messmer)	**1** 2 3 4	1 2 **3** 4
Treatment	Antihistamines, Corticosteroids, Adrenaline (intramuscular)	Antihistamines, Corticosteroids, Adrenaline (intramuscular)
Accompanying factors <24h before injection	consumption of muesli bars: already in the past with allergic symptoms; alcohol, stress	taking non-steroidal anti-inflammatory drugs
Diagnostics		
Total IgE	196 kU/l	127 kU/l
Tryptase	3.98 µg/l	5.0 µg/l
PT results	negative	negative

	Person 1 Substance	Person 1 %CD63+ cells	Person 2 Substance	Person 2 %CD63+ cells
BAT results (positive values)	DMG-PEG 2000* (1:2500)	75,0	DMG-PEG 2000* (1:2500)	10,0
	DMG-PEG 2000* (1:500)	88,0	Comirnaty (1:100)	60,0
	DMG-PEG 2000* (1:100)	17,0	Comirnaty (1:10)	25,0
	PEG** (1:100)	23,0		

* Dimyristoylglycerol-polyethylene glycol 2000
** Polyethylene glycol 2000

**Suppl. Table S4** Comparison of in-vitro diagnostics		Group A*		Group B**	
		Number (n)	Percent (%)	Number (n)	Percent (%)
Total IgE	≤ 100 kU/l	37	84,1	12	75,0
	≥ 100 kU/l	7	15,9	3	18,8
	not tested	0	0,0	1	6,2
Tryptase	≤11.4 µg/l	41	93,2	15	93,8
	≥ 11.4 µg/l	3	6,8	0	0,0
	not tested	0	0,0	1	6,2
Latex	≤ 0.10 kU/l	27	61,4	7	43,8
	≥ 0.10 kU/l	0	0,0	0	0,0
	not tested	17	38,6	9	56,2

* allergic reaction to SARS-CoV2 vaccine
** allergic reaction to SARS-CoV-2 vaccine component / other medical products

**Suppl. Table S5** Symptoms after injection – after diagnostics		Group A*		Group B**	
		Number	Percent	Number	Percent
Symptom occurrence	yes	25	73,5	8	72,7
	no	9	26,5	3	27,3
	total	34	100,0	11	100,0
Airways	Hoarseness	15	41,7	0	0
	Tightness in the throat	3	8,3	0	0
	Persistent dry cough	8	22,2	0	0
	Swelling of the upper airways	4	11,1	0	0
	Stridor (inspiratory)	5	13,9	0	0
	None of the above	1	2,8	8	100,0
Breathing	Rhinitis	1	3,8	0	0
	Breathing difficulties without rales / stridor	2	7,7	0	0
	Shortness of breath / breathing difficulties	2	7,7	0	0
	Bilateral rattling noises / rales	1	3,8	0	0
	None of the above	20	76,9	8	100,0
Shortness of breath Details	Tachypnoea	2	7,1	0	0
	Increased use of the auxiliary muscles	2	7,1	0	0
	Retractions	1	3,6	0	0
	Grunting / abrupt breathing	1	3,6	0	0
	None of the above	22	78,6	8	100,0
Cardiovascular system	Documented hypotension	1	4,0	0	0
	Shock	1	4,0	0	0
	None of the above	23	92,0	8	100,0
Shock Details	Clouding of consciousness	1	4,0	0	0
	No shock	24	96,0	8	100,0
Skin / mucous membrane	Urticaria limited to injection site	1	3,1	1	11,1
	Generalized tingling sensation	3	9,4	0	0
	Generalized itching without rash	3	9,4	1	11,1
	Localized angioedema not at the injection site	3	9,4	0	0
	Generalized erythema	4	12,5	1	11,1
	Generalized urticaria	3	9,4	1	11,1
	None of the above	15	46,9	5	55,6
Other symptoms	Swollen lymph nodes	1	1,9	0	0
	Headache	11	21,2	2	15,4
	Muscle pain and weakness	11	21,2	4	30,8
	Fever	4	7,7	2	15,4
	Vomiting	2	3,8	0	0
	Nausea	5	9,6	0	0
	Abdominal cramps	1	1,9	0	0
	Other symptoms	16	30,8	4	30,8
	None of the above	1	1,9	1	7,7

* allergic reaction to SARS-CoV2 vaccine
** allergic reaction to SARS-CoV-2 vaccine component / other medical product

Suppl. Table S6
Components of SARS-CoV-2 vaccines used in Germany, mRNA-based (as of 03/2024)

Type of vaccine	mRNA vaccine (embedded in lipid nanoparticles)		
Designation	COMIRNATY®<br>(BioNTech-Pfizer)<br>Omikron XBB.1.5 3µg concentrate<br>at age: 6 months - 4 years	COMIRNATY®<br>(BioNTech-Pfizer)<br>Omikron XBB.1.5 10µg injection dispersion<br>at age: 5 - 11 years	COMIRNATY®<br>(BioNTech-Pfizer)<br>Omikron XBB.1.5 30µg injection dispersion<br>at age: 12+ years
Active ingredient	BNT162b2 (=Tozinameran)	BNT162b2 (=Tozinameran)	BNT162b2 (=Tozinameran)
Cap color	reddish brown	blue	grey
Authorization	31.08.2023	31.08.2023	31.08.2023
Additives	((4-Hydroxybutyl)azanediyl)bis(hexane-6,1-diyl)bis(2-hexyldecanoate) (ALC-0315)<br>2-[(polyethylene glycol)-2000]-N,N-ditetradecylacetamide (ALC-0159)<br>Colfosceril stearate (DSPC)<br>Cholesterol<br>Sucrose<br>Water for injection purposes		
	Trometamol<br>Trometamol hydrochloride	Potassium chloride<br>Potassium dihydrogen-phosphate<br>Sodium chloride<br>Sodium monohydrogen-phosphate 2H$_2$0<br>Sodium hydroxide<br>Hydrochloric acid	Potassium chloride<br>Potassium dihydrogen-phosphate<br>Sodium chloride<br>Sodium monohydrogen-phosphate 2H$_2$0<br>Sodium hydroxide<br>Hydrochloric acid

Suppl. Table S7
Components of SARS-CoV-2 vaccines used in Germany, protein-based (as of 03/2024)

Type of vaccine	protein-based vaccine
Designation	Nuvaxovid (Novavax)<br>Omikron XBB.1.5 5µg<br>injection dispersion<br>at age: 12+ years
Active ingredient	NVX-CoV2373 spike protein* of SARS-CoV-2 (produced by recombinant DNA technology using a baculovirus expression system in an insect cell line from Sf9 cells of the species Spodoptera frugiperda), adjuvant Matrix-M per 0.5 ml dose contains fraction-A (42.5 µg) + fraction-C (7.5 µg) of Quillaja Saponaria Molina extract
Cap color	blue
Authorization from the European Commission	31.10.2023
Additives	Polysorbate 80<br>Phosphatidylcholine (including all-rac-α-tocopherol)<br>Cholesterol<br>Water for injection purposes<br>Potassium chloride<br>Potassium dihydrogen phosphate<br>Sodium chloride<br>Disodium hydrogen phosphate-7 H_2O and -2 H_2O<br>Sodium dihydrogen phosphate 1 H_2O<br>Sodium hydroxide (for pH adjustment)<br>Hydrochloric acid (for pH adjustment)

Supplementary Figures

Fig. S1 Illustration of the workflow and the results of allergological diagnostics

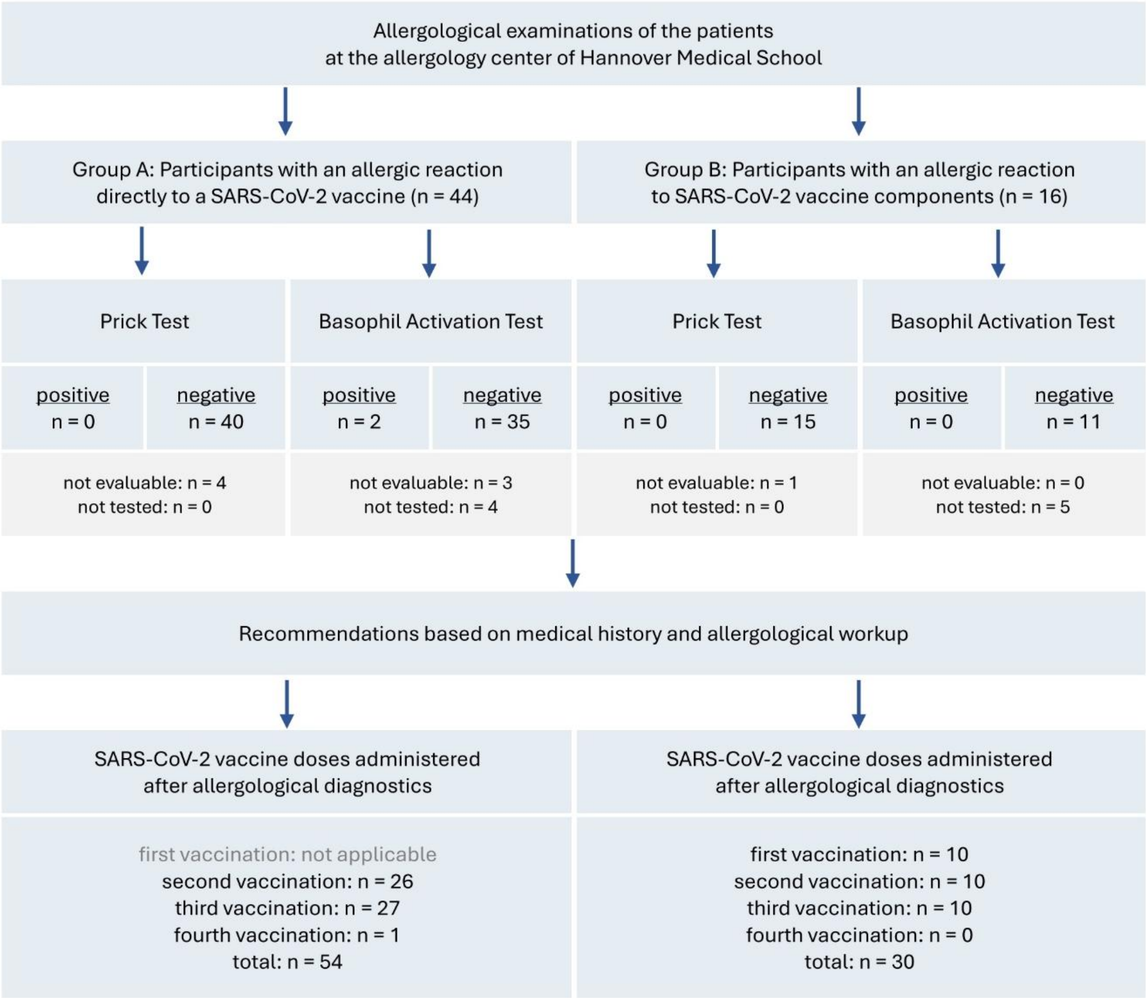

Fig. S2 SARS-CoV-2 vaccine doses administered after diagnostics

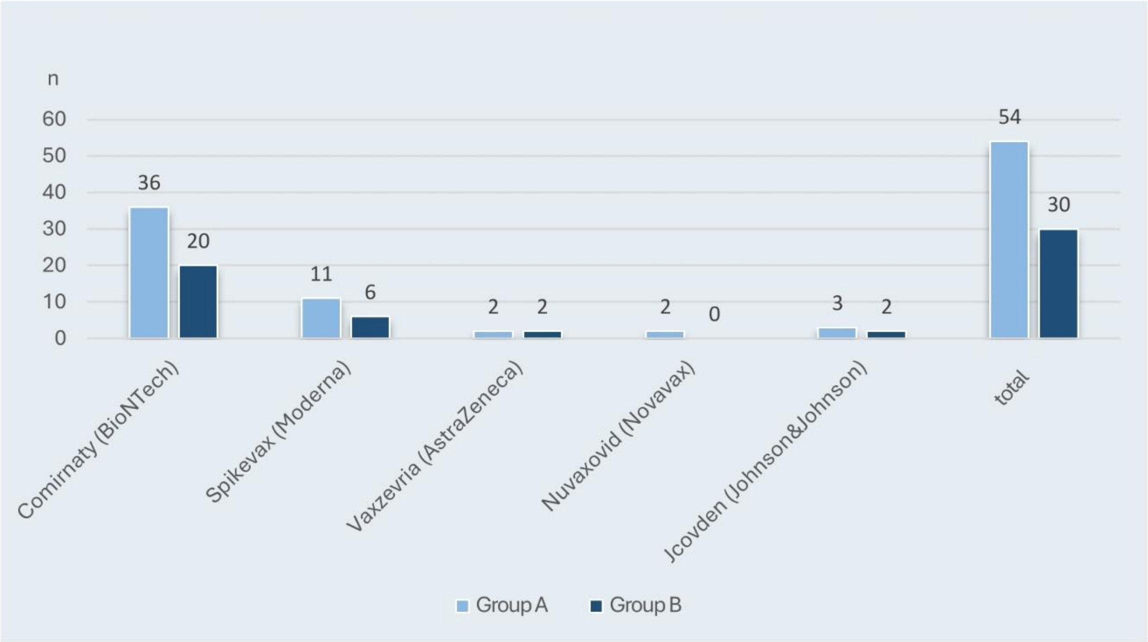

Supplement: Supplemental material [file allergologieselect-10-063-S01.pdf]
